# 17β-Estradiol Effects in Skeletal Muscle: A ^31^P MR Spectroscopic Imaging (MRSI) Study of Young Females during Early Follicular (EF) and Peri-Ovulation (PO) Phases

**DOI:** 10.3390/diagnostics14030235

**Published:** 2024-01-23

**Authors:** Jimin Ren, Luis Rodriguez, Talon Johnson, Anke Henning, Yasin Y. Dhaher

**Affiliations:** 1 Advanced Imaging Research Center, University of Texas Southwestern Medical Center, Dallas, TX 75390, USA; talon.johnson@utsouthwestern.edu; 2 Department of Radiology, University of Texas Southwestern Medical Center, Dallas, TX 75390, USA; 3 Department of Bioengineering, University of Texas at Dallas, Richardson, TX 75080, USA; luis.rodriguez2@utsouthwestern.edu; 4 Department of Physical Medicine and Rehabilitation, University of Texas Southwestern Medical Center, Dallas, TX 75390, USA; 5 Peter O’Donnell Jr. Brain Institute, University of Texas Southwestern Medical Center, Dallas, TX 75390, USA

**Keywords:** phosphocreatine, estrogen, skeletal muscle, metabolism, magnesium, 31P MRS, 7T

## Abstract

The natural variation in estrogen secretion throughout the female menstrual cycle impacts various organs, including estrogen receptor (ER)-expressed skeletal muscle. Many women commonly experience increased fatigue or reduced energy levels in the days leading up to and during menstruation, when blood estrogen levels decline. Yet, it remains unclear whether endogenous 17β-estradiol, a major estrogen component, directly affects the energy metabolism in skeletal muscle due to the intricate and fluctuating nature of female hormones. In this study, we employed 2D ^31^P FID-MRSI at 7T to investigate phosphoryl metabolites in the soleus muscle of a cohort of young females (average age: 28 ± 6 years, *n* = 7) during the early follicular (EF) and peri-ovulation (PO) phases, when their blood 17β-estradiol levels differ significantly (EF: 28 ± 18 pg/mL vs. PO: 71 ± 30 pg/mL, *p* < 0.05), while the levels of other potentially interfering hormones remain relatively invariant. Our findings reveal a reduction in ATP-referenced phosphocreatine (PCr) levels in the EF phase compared to the PO phase for all participants (5.4 ± 4.3%). Furthermore, we observe a linear correlation between muscle PCr levels and blood 17β-estradiol concentrations (r = 0.64, *p* = 0.014). Conversely, inorganic phosphate Pi and phospholipid metabolite GPC levels remain independent of 17β-estradiol but display a high correlation between the EF and PO phases (*p* = 0.015 for Pi and *p* = 0.0008 for GPC). The robust association we have identified between ATP-referenced PCr and 17β-estradiol suggests that 17β-estradiol plays a modulatory role in the energy metabolism of skeletal muscle.

## 1. Introduction

The secretion of 17β-estradiol, a key estrogen hormone, undergoes natural variations throughout the menstrual cycle in young women. These fluctuations, while integral to the female reproductive system, also exert significant influence over various organs, including skeletal muscle, the largest in the human body (comprising approximately 37% of the female body mass). Several estrogen receptors (ER) have been found in skeletal muscle, including the membrane-bound GPER and nuclear-type ER-α/β, both of which are activated upon binding with estrogen molecules [1,2,3,4,5]. During or before menstruation, when blood estrogen levels decrease, many women frequently experience increased fatigue and diminished energy levels [6,7]. Additionally, estrogen deficiency, often seen in menopausal women, has been linked to a range of musculoskeletal and neuromuscular disorders, such as sarcopenia, osteoporosis, frailty, obesity, dementia, atherosclerosis, metabolic syndrome, and type 2 diabetes, with reported benefits when endogenous estrogen levels rise or exogenous estrogen is supplemented [2,8,9,10,11,12,13,14,15,16].

Despite these observations, a comprehensive understanding of the effects of 17β-estradiol on the energy metabolism in skeletal muscle remains elusive due to the complex and fluctuating nature of sex hormones throughout the menstrual cycle [4,14]. In our study, we utilized advanced 2D ^31^P FID-MRSI (31-Phosphorus Magnetic Resonance Spectroscopic Imaging) at the ultrahigh magnetic field 7T to investigate alterations in phosphoryl metabolites within the soleus muscle of young females during two distinct menstrual phases: the early follicular (EF) and peri-ovulation (PO) phases. Importantly, these phases were selected for their significant disparities in blood 17β-estradiol levels, while other potentially interfering hormones, such as progesterone and parathyroid hormone (PTH), remained consistently low [14].

The soleus muscle was chosen as the focal point of our study due to its pivotal role in various weight-bearing activities, such as walking, running, and jumping, all of which are substantially affected by sarcopenia and osteoporosis [17,18], the two most prevalent musculoskeletal disorders that share a common dysregulation pattern in females, marked by declining estrogen bioavailability [18,19]. Metabolically, the soleus muscle, composed of approximately 78% type I fibers and well-perfused by blood, is considered to be fatigue-resistant and oxidative in energetics. Furthermore, its favorable volume-based shimming properties, including a large size and inner location, make it particularly suitable for localized MRS studies. Consequently, the soleus muscle promises an intrinsically high detection sensitivity and spectral resolution, making it an ideal option for capturing subtle metabolic changes resulting from hormonal fluctuations during the menstrual cycle.

Recent ^31^P MRS studies have hinted at the influence of gender and menopausal status in the mitochondrial energy metabolism, albeit primarily in the context of the brain, another organ rich in estrogen receptors [20,21]. For instance, using 3T ^31^P MRSI, researchers found lower ATP-referenced phosphocreatine (PCr) levels in the brains of postmenopausal women compared to men [22]. In animal models, estrogen withdrawal was observed to significantly increase the nucleoside ATP/Pi ratio in estrogen-dependent human breast cancer xenografts, while dietary creatine supplementation inhibited the growth of various tumors by increasing PCr levels without affecting ATP content [23,24]. Remarkably, to the best of our knowledge, the impact of endogenous 17β-estradiol on PCr in skeletal muscle has not yet been explored using 31P MRSI techniques.

## 2. Materials and Methods

### 2.1. Cohort Characterization, Protocol Approvals, and Consent

This study was approved by the Institutional Review Board of The University of Texas Southwestern Medical Center on 24 August 2020 (protocol number STU-2020-0744). Seven healthy eumenorrheic females participated in this study, with no history of lower limb disorder, age 28 ± 6 years (range 21–38), BMI 25 ± 4 kg/m^2^, heart rate 68 ± 22 bpm, and blood oxygen saturation 99 ± 1%. Exclusion criteria included a history of musculoskeletal or orthopedic injury of the spine, hip, knee, ankle, or foot; history of neurological injury or disease of the peripheral or central nervous system; current smoking habit; history of disordered eating, stress fracture, connective tissue disorder (Marfan syndrome, Ehlers–Danlos disease), or menstrual dysfunction (primary or secondary amenorrhea, oligomenorrhea, anovulatory cycles, or polycystic ovarian disease); current or prior pregnancy; use of an oral contraceptive within the previous 6 months; or use of an injectable or implantable contraceptive. All participants were scanned in both EF and PO phases according to their menstrual cycles. Informed and written consents were obtained from all subjects prior to MRI scans.

### 2.2. MRS Protocol

All subjects were positioned feet-first and supine in the MRI scanner (7T Achieva, Philips Healthcare, Best, The Netherlands), with the calf muscle of the non-dominant leg positioned in the center of the RF coil (Philips Healthcare, Best, The Netherlands). The leg position was secured by Velcro straps with a thick pad in between for cushioning, and the subjects were asked to keep still and not to make muscle excursions during the scan. The RF coil was a half-cylinder-shaped partial volume, double-tuned ^1^H/^31^P quadrature transmit/receive coil consisting of two tilted, partially overlapping 10 cm loops, with a solid base that could be firmly attached to the scanner table. Axial and sagittal T2-weighted turbo spin echo images were acquired for planning the ^1^H-based Bo shimming (by second order pencil-beam projection method, with the shimming volume located in the soleus muscle) and the ^31^P MRSI acquisition data matrix.

^31^P MR spectra were acquired with a 2D FID-MRSI sequence using a block pulse at TR 1.0 s, TE 0.48 ms, B_1_ 59 μT, flip angle 55°, effective excitation bandwidth 3.2 kHz, receiver bandwidth 8 kHz, in-plane resolution of 8 × 7.5 mm^2^, k-space acquisition weighting (α = 1.7 and β = 1.0), elliptic k-space sampling with 4 k points zero-filled to 8 k prior to Fourier transformation, data matrix (RL × AP) = 12 × 6 reconstructed to 15 × 8, field-of-view FOV (RL × AP) = 120 × 60 mm^2^, nominal slice thickness 30 cm, number of average NA = 16, and scan time 7 min. A non-localized, fully relaxed ^31^P spectrum was also acquired from each subject with a pulse-acquire sequence at TR = 25 s and NA = 8.

### 2.3. Data Analysis

The time-domain ^31^P FID data were post-processed (zero-filling, apodization, Fourier transformation, and zero- and first-order phasing) using the scanner software (SpectroView R5.7, Philips Healthcare). To correct Bo inhomogeneity, frequency-domain ^31^P spectra from selected soleus voxels were summed after being individually aligned to PCr at 0 ppm. To reduce potential B1 inhomogeneity, only central soleus voxels within the shimming volume with a comparably high-intensity signal were selected. The summed voxel spectra then underwent lineshape fitting analysis using an in-house program written in Matlab 2021b (MathWorks, Natick, MA, USA) [25,26,27]. Typically, all ^31^P peaks of interest were fitted by a single Gaussian lineshape model except Pi, which was deconvoluted into extra- and intracellular components, (Pi(ex) and Pi(in)), and the mixed signal in the region −7.5–−9.5 ppm, which was deconvoluted into α-ATP and NAD, using two Gaussian lineshapes at different chemical shifts. Prior knowledge of the peak chemical shifts was included as a soft constraint [25]. The PCr map representing an interpolation to the same resolution as the T2-weighted image was generated using SpectroView by bilinear interpolation from the nearest voxels. Voxel-summed 31P MR spectra were obtained, respectively, for the EF and PO phases over all subjects (*n* = 7). All signals were normalized with respect to γ-ATP in integral, without T1 correction for a partial saturation effect.

### 2.4. Evaluation of Intracellular pH

The intracellular pH was evaluated from the chemical shift of the Pi resonance (δ_Pi_) according to the Henderson–Hasselbalch equation, as published previously [21]:

pH = pK_a_ + log_10_[(δ_Pi_ − δ_a_)/(δ_b_ − δ_Pi_)] (1)

where the reaction H_2_PO_4_^−^ ↔ H^+^ + HPO_4_^2−^ deprotonation constant pK_a_ = 6.73 and the ^31^P limiting shifts δ_a_ = 3.275 ppm (for the acidic protonated species H_2_PO_4_^−^) and δ_b_ = 5.685 ppm (for the basic deprotonated species HPO_4_^2−^) were used in the data analysis.

### 2.5. Evaluation of Intracellular Mg^2+^

The intracellular free Mg^2+^ concentration was evaluated from the chemical shift of β-ATP resonance (δ_β_) according to the following equation:

[Mg^2+^] = *k_d_* (δ_β_ − δ_ATP_)/(δ_MgATP_ − δ_β_) (2)

where the reaction MgATP ↔ Mg^2+^ + ATP disassociation constant *k_d_* = 0.05 mM (or pk_d_ = 4.30) and the limiting shifts δ_MgATP_ = −15.74 ppm (for the 1:1 complex MgATP) and δ_ATP_ = −18.58 ppm (for the free ATP species) were used in the data analysis.

### 2.6. Analysis of 17β-Estradiol

Circulating 17β-estradiol concentrations were measured in serum. Briefly, venipuncture was performed at the antecubital area of the arm. Whole blood was collected with a red top, vacutainer serum collection tube (Becton Dickinson, Franklin Lakes, NJ, USA), processed according to the manufacturer’s recommendations, aliquoted, and stored at −80 °C for future analysis. Frozen serum samples were then sent to a clinical laboratory service (Medfusion, Lewisville, TX, USA) to confirm the self-reported menstrual cycle phase of the subject.

### 2.7. Statistical Analysis

Data are reported as mean ± standard deviation, and a *p*-value ≤ 0.05 is statistically significant. A two-sample one-tailed *t*-test was performed using the Matlab’s function *ttest2* for the alternative hypothesis that the EF and PO phases have unequal means of ^31^P signal intensities. Post hoc power analysis was performed using the Matlab’s function *sampsizepwr* to compute the statistical power achieved with the experimental sample size (*n* = 7) and the means and variances of actual measurements obtained in this study.

## 3. Results

### 3.1. Voxel ^31^P MRSI Spectra

**Figure 1:**
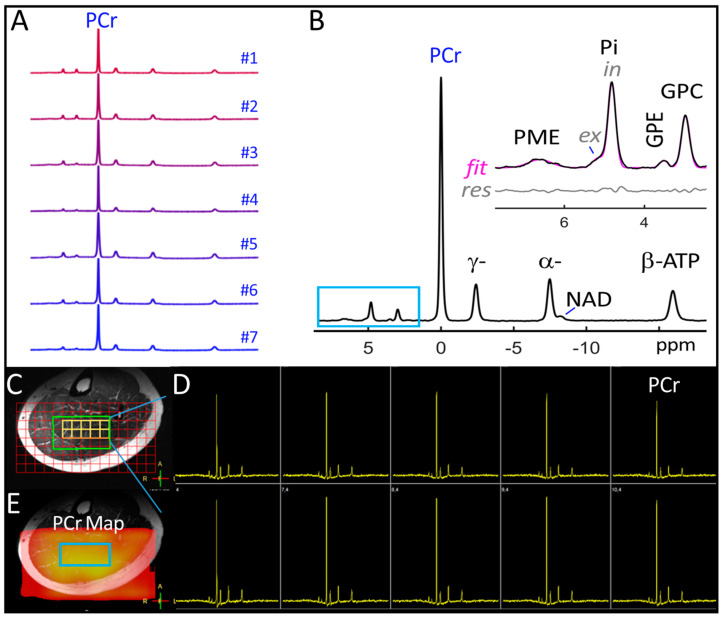
(**A**) 7T ^31^P spectra acquired by 2D MRSI from the soleus muscle in seven females. (**B**) Group-summed 31P spectrum. (**C**) 2D ^31^P MRSI matrix showing the placement of voxels (red) and the shimming box (green) over an axial T2w MR image. (**D**) Screenshot of voxel ^31^P spectra in the soleus region of interest (yellow matrix, (**C**)). (**E**) PCr color map reconstructed from voxel ^31^P spectra.

The 7T ^31^P MRSI spectra acquired from the selected soleus voxels exhibited excellent spectral resolution and SNR, as evident in spectra from individual subjects (Figure 1A) and the cohort-summed spectrum (Figure 1B). High-intensity, well-defined signals include Pi(in), GPC, PCr, α-, β-, and γ-ATP, which can be easily detected from single voxels (Figure 1C,D). Low-intensity signals that can be reliably measured only in voxel-summed spectra include Pi(ex), GPE, PME (a composite of various metabolites primarily constituted of PE, PC, and sugar phosphates), and NAD (a mixture of both oxidized and reduced forms). Notably, among these measurable metabolites, PCr exhibited the highest detection sensitivity, and its distribution across the axial plane could be spatially mapped (Figure 1E). Table 1 summarizes the chemical shifts and ATP-referenced signal intensities for all measurable 31P peaks in the soleus muscle at rest.

### 3.2. EF vs. PO ^31^P Spectra

**Figure 2:**
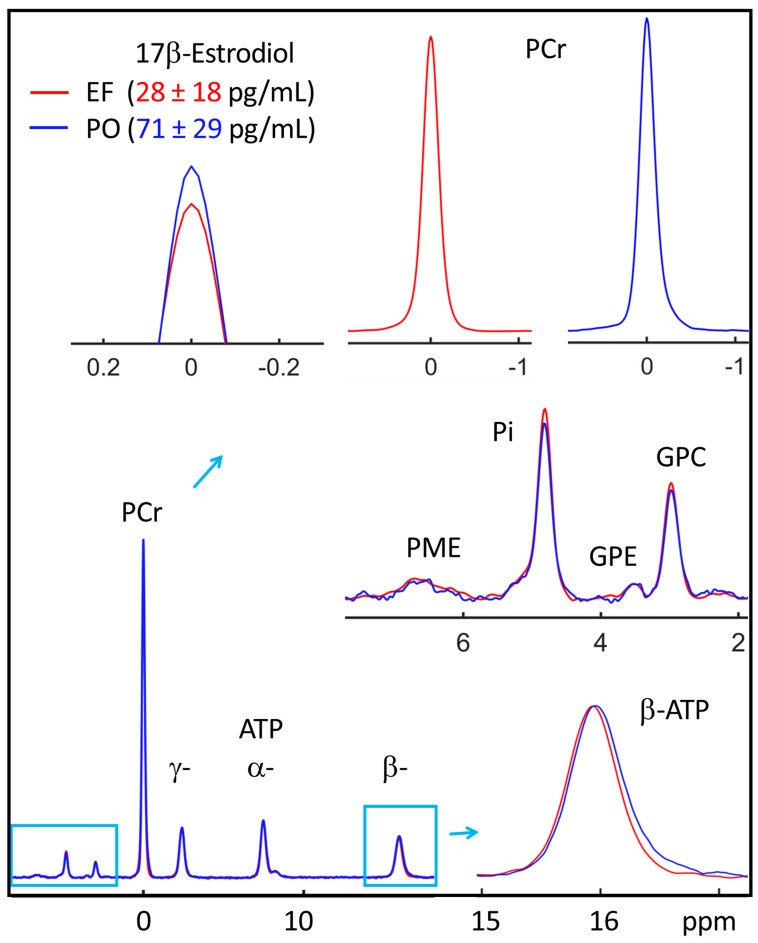
Comparison of group-averaged voxel ^31^P MR spectra acquired from the soleus muscle during EF (red) and PO (blue) phases (*n* = 7). Insets: enlarged signals showing EF and PO difference in PCr signal intensity (top) and β-ATP chemical shift (bottom). No EF and PO difference was seen in the chemical shift at Pi with respect to PCr (at 0 ppm).

Figure 2 compares the group-averaged ^31^P spectra acquired during the EF and PO phases. A notable difference is evident in the PCr signal, which exhibited a 6% decrease in the EF phase (red trace), as compared to the PO phase (blue trace, top, Figure 2). Their difference in mean PCr-to-γ-ATP ratio (EF 3.82 ± 0.18 vs. PO 0. 42 ± 0.18, Table 1) is statistically significant (*p* = 0.042 and h = 1). Post hoc power analysis of these data showed that, for the alternative hypothesis that the EF phase has a significantly lower PCr level than the PO phase, the achieved statistic power is 0.91 with the sample size of *n* = 7.

Additionally, a downfield peak shift was observed at β-ATP (by 0.02 ppm), indicative of a higher free Mg concentration (0.06 mM) in the EF phase relative to the PO phase. In contrast, intracellular pH remained remarkably similar between these two phases (ΔpH < 0.01 unit), as evidenced by the negligible change in chemical shift at Pi(in) (0.006 ppm). No significant difference was detected for the low-magnitude signals PME, Pi(ex), GPE, and NAD.

**Figure 3:**
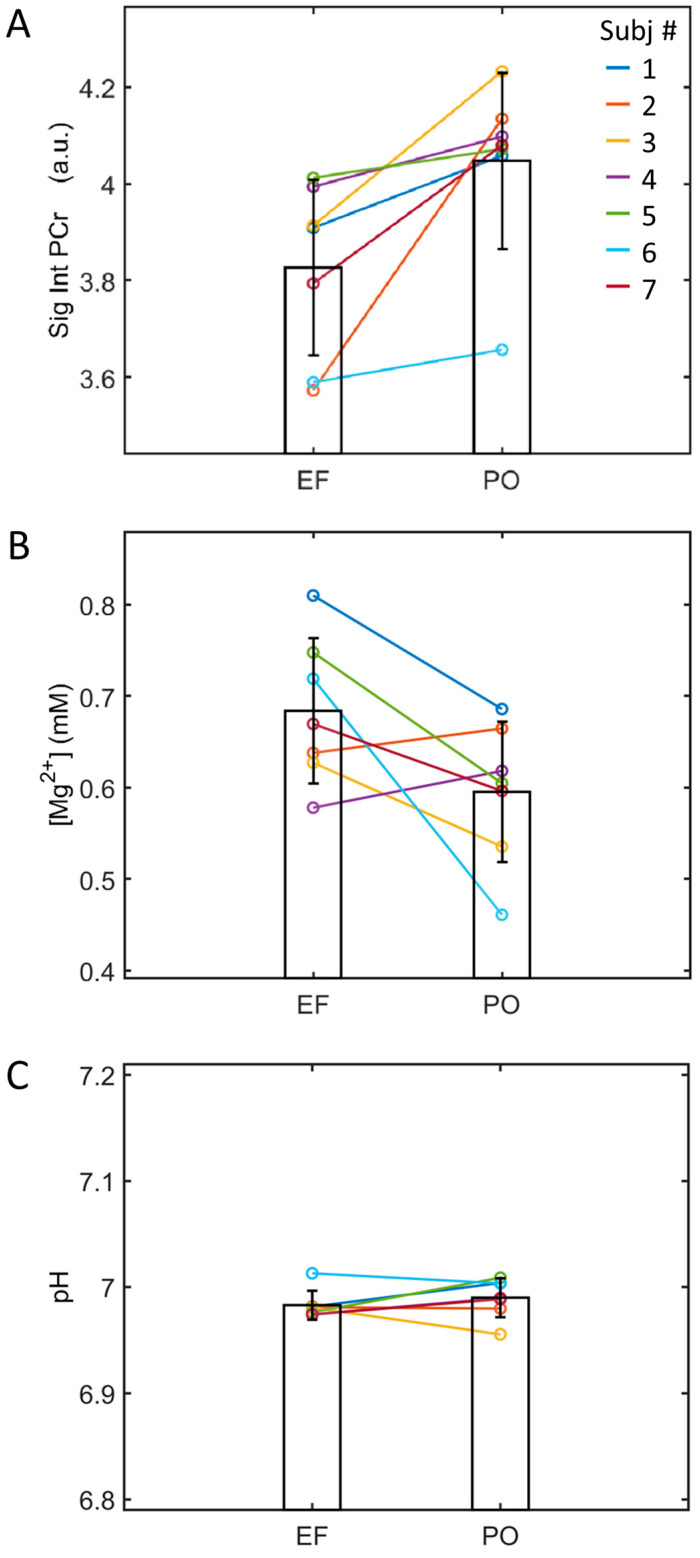
Comparison between EF and PO in PCr 31P signal intensities (**A**), free Mg^2+^ concentrations (**B**), and (**C**) pH for individuals.

Figure 3 represents individual measurement results for the EF and PO phases. The decline in PCr during the EF phase compared to the PO phase was consistent across all seven participants, ranging from 1.5% to 13.5% (averaging 5.4 ± 4.3%, Figure 3A). In five out of the seven subjects, a higher cytosolic free Mg concentration was observed during the EF phase compared to the PO phase, with a group average of 0.68 ± 0.08 mM in the EF phase versus 0.60 ± 0.08 mM in the PO phase (Figure 3B). The difference in Mg levels was nearly significant but did not reach statistical significance, with a *p*-value of 0.542. The pH levels exhibited minimal variation between these two phases, averaging 6.983 ± 0.014 in the EF phase and 6.990 ± 0.018 in the PO phase (Figure 3C).

### 3.3. EF and PO Difference in 17β-Estradiol

Serum measurements confirmed a substantial decrease in 17β-estrogen concentration during the EF phase compared to the PO phase (28 ± 18 vs. 71 ± 30 pg/mL, *p* = 0.021). Notably, the levels of serum 17β-estrogen displayed considerable heterogeneity among individuals within this cohort, ranging from less than 10 to 62 pg/mL during the EF phase and from 36 to 116 pg/mL during the PO phase. This wide range of values allows for a more comprehensive exploration of the dependence of ^31^P measurements on 17β-estrogen.

### 3.4. Blood 17β-Estrogen and Soleus ^31^P MRS Correlation

**Figure 4:**
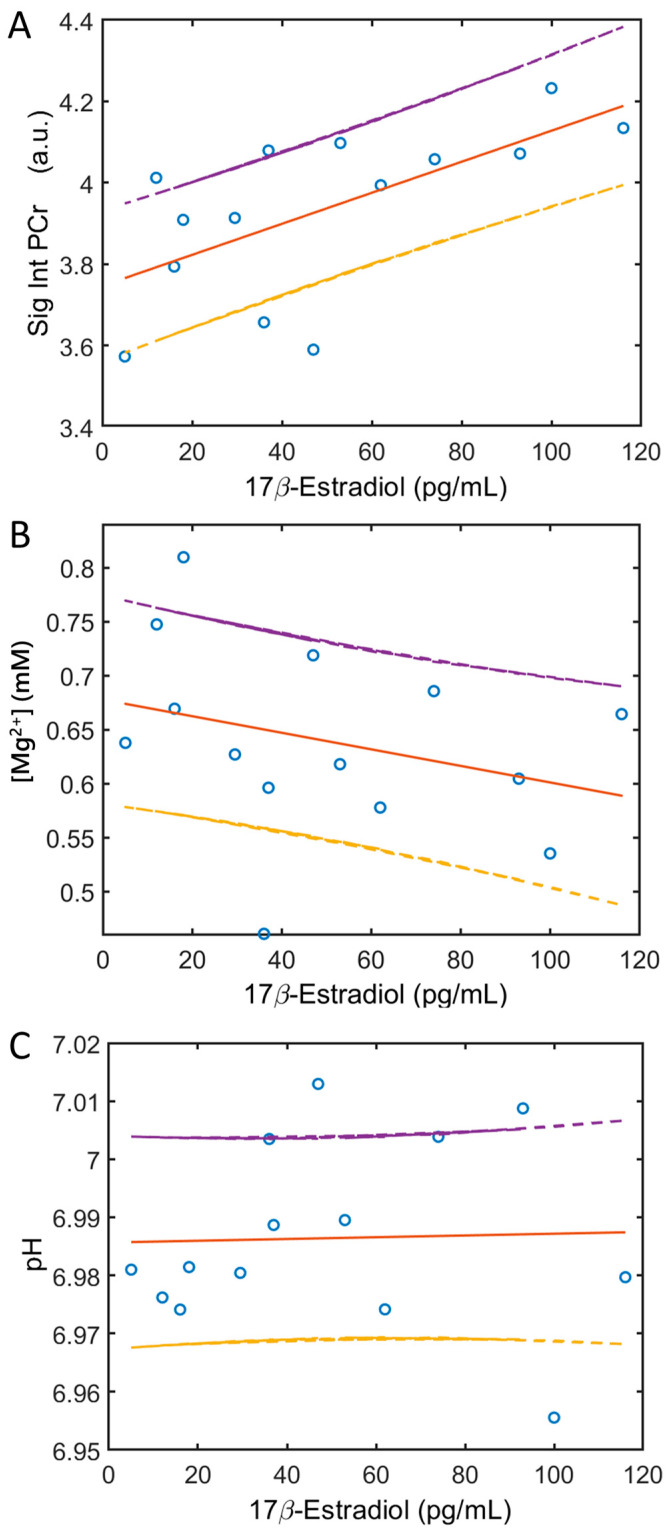
Linear correlation of the blood 17β-estradiol concentrations with PCr ^31^P signal intensities ((**A**), r = 0.64, *p* = 0.014), free Mg^2+^ concentrations ((**B**), r = −0.30, *p* = 0.29), and pH ((**C**), r = 0.03, *p* = 0.91) for individual subjects in the EF and PO phases. Solid lines show the fitted data and dashed lines show ± 1 unit of standard deviation.

Table 2 summarizes the results of the linear correlation analysis. A significant linear correlation was found between blood 17β-estrogen levels and muscle PCr signal intensities (r = 0.638, *p* = 0.014, Figure 4A). Notably, the ATP-referenced PCr signal exhibited a tendency to increase with rising 17β-estrogen levels (with an intercept of 3.746 and a slope of 0.004). Conversely, an inverse trend was observed for intracellular free Mg (Figure 4B); however, this correlation did not attain statistical significance (r = −0.304, *p* = 0.290). Importantly, no significant correlation was detected between blood 17β-estrogen levels and muscle intracellular pH (Figure 4C).

### 3.5. EF and PO Metabolite Correlation

**Figure 5:**
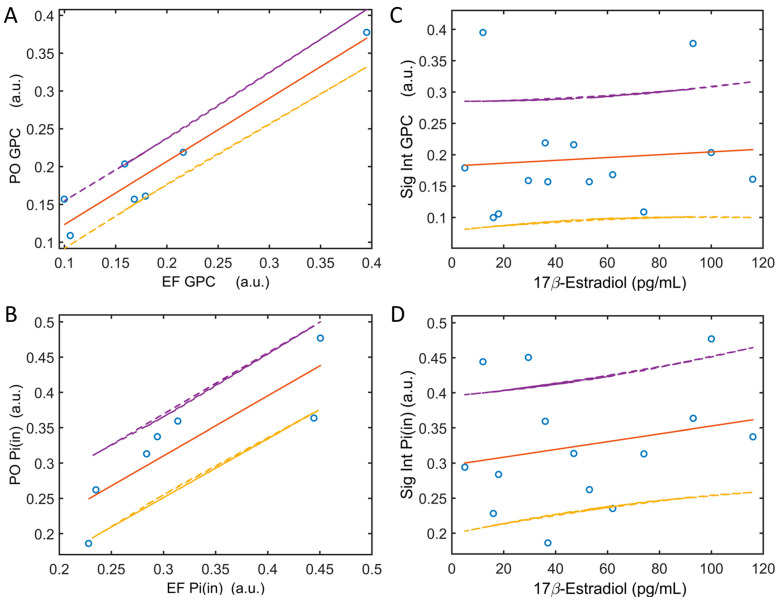
Linear correlation of EF and PO data in 31P signal intensities of GPC ((**A**), r = 0.956, *p* = 0.0008) and Pi(in) ((**B**), r = 0.850, *p* = 0.015). Linear correlation of blood 17β-estradiol levels with 31P signal intensities of GPC ((**C**), r = 0.088, *p* = 0.766) and Pi(in) ((**D**), r = 0.280, *p* = 0.332). Note that there are strong signal correlations between EF and PO in GPC and Pi(in) 31P signals (**A**,**B**) when these signals appear to be independent of 17β-estradiol.

Within this subject cohort, the 31P signal intensities of GPC and Pi(in) (Figure 1A) exhibited a broad range of values (Figure 5). A robust linear correlation with a unity slope was almost evident for both GPC (r = 0.956, *p* = 0.0008) and Pi(in) (r = 0.850, *p* = 0.015) when comparing the EF and PO phases (Figure 5A,B). However, neither GPC nor Pi(in) displayed a linear correlation with 17β-estrogen levels (Figure 5C,D). Instead, it appears that body mass index (BMI) is a noteworthy factor influencing GPC variations among individuals, as indicated by the results of the linear correlation analysis (r = 0.708, *p* = 0.075). BMI also appears to affect PME and extracellular inorganic phosphate, with correlation coefficients of r = 0.632 (*p* = 0.127) for PME and r = 0.679 (*p* = 0.093) for Pi(ex).

## 4. Discussion

### 4.1. Major Findings

To the best of our knowledge, this study represents the first quantitative investigation into the influence of the sex hormone 17β-estradiol on the energy metabolism within the soleus muscle utilizing localized ^31^P MRSI. The results demonstrated a significant linear correlation between the high-energy metabolite PCr and blood 17β-estradiol levels in young females. Particularly, the γ-ATP-referenced PCr levels within the soleus muscle exhibit a decline during the low-17β-estradiol EF phase in contrast to the high-17β-estradiol PO phase. This finding suggests a potential role for 17β-estradiol in influencing the energy metabolism of skeletal muscle.

### 4.2. Role of PCr in Energy Metabolism

PCr, serving as the immediate buffer for the universal bioenergy currency ATP, is notably abundant in skeletal muscle. This abundance extends to both its overall quantity, considering that skeletal muscle comprises approximately 30–37% of the female body mass, and its PCr-to-ATP ratio, which is notably higher in skeletal muscle (~4.0 [26]) compared to other tissues such as the heart (~2.0 [28,29]), brain (~1.5 [25,27]), kidney and male prostate (~1.0 [30,31,32,33]), and liver (<0.2 [34]). These characteristics underscore the pivotal role of PCr in maintaining energy homeostasis within skeletal muscle, particularly in responding to rapidly fluctuating energy demands.

A heightened PCr store implies an increased capacity for ATP buffering, facilitated by the creatine kinase (CK) reaction. A decline in PCr levels may negatively affect muscle performance, thus contributing to the fatigue and physical stress commonly associated with menstrual and premenstrual syndromes. Conversely, the finding of elevated PCr levels during the PO phase compared to the EF phase aligns with the idea that rising estrogen levels, whether endogenously or through replacement therapy, could enhance physical energy and improve productivity for many females throughout their reproductive and postmenopausal stages [16].

Indeed, the impacts of a deficiency in CK substrates on physiological function are well-documented. Conditions like brain creatine deficiency syndrome have been established to be associated with epilepsy and movement disorders [35,36,37]. Additionally, CK substrate deficiency can exert adverse effects on mitochondrial function, particularly within the context of energy production in skeletal muscle [38].

In cytosolic CK knockout mice, a reduced breakdown in PCr to support ATP regeneration was found to be associated with a delayed muscle relaxation after repeated muscle contractions [39].

### 4.3. Correlation between PCr and 17β-Estradiol

The increased PCr in the soleus muscle in the PO phase relative to the EF phase was attributed to the effect of increasing 17β-estradiol concentrations in the blood (Figure 3 and Figure 4A), as suggested by the linear correlation between these two measurements (*p* < 0.05, Figure 5A). We excluded the possibility that the observed PCr change from the EF to PO phase is due to prior exercise or incident muscle excursion during the scan, given that all subjects in this cohort were well-screened for physical exercise 48 h prior to the MRI visit and remained at rest during the scan, with continuous monitoring. A high detection sensitivity and spectral resolution at 7T is a key factor in revealing small changes. A previous ^31^P MRS study reported an observation of change in the brain high-energy metabolites upon visual stimulation [40], though another study at 3T did not see significant change at the detection threshold in the brain (~5% [41]). It should be noted that, in standard experimental conditions, SNR increased supralinearly with field strength (SNR~B0^1.65^, [42]), implying that the MRS detection sensitivity at 7T is fourfold higher than that at 3T. Furthermore, in skeletal muscle the PCr detection sensitivity is about one order of magnitude higher than in the brain under comparable conditions, mainly due to PCr’s intrinsically higher concentrations (skeletal muscle, ~35 mM, vs. the brain, ~3.5 mM). All these factors contribute to the exceptional sensitivity of 7T ^31^P MRS in capturing subtle metabolic changes in skeletal muscle during different phases of the menstrual period.

Given the significant correlation observed between blood 17β-estradiol and muscle PCr levels in the current study (Figure 3A and Figure 4A), and considering that 17β-estradiol is the only primary hormone undergoing a dramatic elevation during the transition from the EF to PO phase, as established in a previous study profiling female hormones in the menstrual cycle [43], it is likely that 17β-estradiol levels mediate the relationship between the menstrual cycle phase and muscle energy metabolism in young females. A potential mechanism for this relationship is discussed in the following section.

### 4.4. Acting Sites of 17β-Estradiol

17β-estradiol, as a hydrophobic steroid hormone, is unlikely to be engaged in direct chemical interactions with water-soluble energy metabolites in the cytosol. However, its influence on the energy system may result from its binding to receptors located in various cellular compartments, including the nucleus (where the majority of ERs are concentrated), mitochondria, and the cell membrane (comprising a smaller pool of ERα and ERβ receptors, ~5–10%) [44]. PCr is vital for ATP regeneration within mitochondria, and the regulation of mitochondrial function by estradiol has been the topic of recent reviews [45,46,47]. Dysregulation of this estrogen signaling system has been proposed as a potential trigger for pathologies such as Alzheimer’s disease in postmenopausal women [48]. Notably, this neurodegenerative condition is associated with a decrease in ATP-referenced PCr levels [49], aligning well with our findings of lower PCr levels during the low-17β-estradiol early follicular phase compared to the high-17β-estradiol peri-ovulation phase (Figure 3A).

### 4.5. Membrane Phospholipids (MPL) Metabolites

Considering the established role of estrogen in preserving cell membrane integrity and reducing oxidative damage [50], it is reasonable to speculate that elevated 17β-estradiol levels might lead to a decrease in the cytosolic products from the degradation of membrane phospholipids (MPL). In the current 31P study, GPC is the sole MPL degradation metabolite with sufficient abundance for reliable measurement (Figure 1A). While GPC levels do display considerable variability among individual subjects and remain highly consistent between two separate visits during both EF and PO phases (Figure 5A), we did not observe any correlation between GPC levels and serum 17β-estradiol levels (Figure 5C).

These findings suggest that the anticipated protective role of 17β-estradiol against membrane oxidation damage does not result in measurable changes in the cleavage of hydrophilic phospholipid head groups in association with cytosolic GPC accumulation. Instead, its protection on membrane integrity may result from its hydrophobic interaction with the fatty acid moiety of MPLs, presumably through the membrane-bound receptors ERα and ERβ located in lipid rafts [47].

### 4.6. pH and Free Mg Measurements

Our investigation did not yield evidence of 17β-estradiol-dependent changes in cellular pH between the EF and PO phases (Figure 3C and Figure 4C). Nevertheless, it is noteworthy that in the majority of the subjects studied (five out of seven), a higher concentration of free Mg^2+^ was observed in the EF phase compared to the PO phase (Figure 3B). While the intracellular [Mg^2+^] appears to be influenced by 17β-estradiol levels, it is not notably strong (Figure 4B) as compared to PCr (Figure 4A).

The role of Mg^2+^ as an essential cofactor in enzymatic ATP hydrolysis activation highlights its critical importance in the realm of energy metabolism and muscle function [51]. Maintaining adequate levels of Mg^2+^ has long been recognized as crucial in the prevention of fatigue and the alleviation of muscle cramps and spasms, issues commonly encountered not only by endurance athletes but also by females dealing with menstrual syndromes [52,53]. Elevated levels of muscle Mg^2+^ in the EF phase may effectively counteract Ca^2+^ to mitigate muscle spasms or twitches resulting from excessive muscle contraction due to Ca^2+^ accumulation. Furthermore, it is noteworthy that natural postmenopausal women typically exhibit lower serum estrogen levels in conjunction with higher magnesium levels when compared to healthy premenopausal women [54]. Additionally, estrogen supplementation has been shown to reduce the hypermagnesuria observed in postmenopausal women [55]. Elevated muscle magnesium levels have also been frequently documented in chronic fatigue syndrome [56], a condition that primarily affects middle-aged individuals and is four-times more prevalent in women than in men [57].

Our current study aligns with these previous findings by demonstrating higher Mg^2+^ levels in the low-17β-estradiol EF phase relative to the high-17β-estradiol PO phase (*p* = 0.054, Figure 3B). Taken together, these results provide compelling evidence for a potential interplay between 17β-estradiol regulation and magnesium metabolism, which may involve complex interactions among bones (where approximately 50% of Mg is stored [58]), kidneys (where regulation of Mg occurs [59]), and skeletal muscle [15,16]. Further research is needed to explore and elucidate the intricate relationships between these factors and their implications for overall women’s health and well-being.

While not the primary focus of this study, our data analysis indicates a tendency of BMI to influence GPC and PME signals, suggesting a potential association between BMI and membrane phospholipid metabolism. This observation is consistent with prior research findings [60,61,62].

A limitation of this preliminary study is its relatively small cohort size, consisting of only seven participants. Nevertheless, our post hoc analysis suggests the generalizability of our key finding that the high-energy PCr pool is reduced in the EF phase compared to the PO phase, supported by a robust statistical power of 0.91, despite the small sample size. To strengthen the validity of our results, further research with a larger cohort is anticipated. Additionally, expanding the scope of this study to investigate other hormones, such as progesterone, which is markedly elevated during the late luteal phase of the menstrual cycle, could provide a more comprehensive understanding of the underlying metabolisms. Furthermore, it would be intriguing to explore the influence of endogenous 17β-estradiol on the glycolytic gastrocnemius muscle in comparison to the more oxidative soleus muscle.

In conclusion, using localized 7T ^31^P MRSI, our study has revealed a decline in ATP-referenced PCr levels during the low-17β-estradiol early follicular phase compared to the high-17β-estradiol peri-ovulation phase. Furthermore, we have demonstrated a significant linear correlation between the ATP-referenced PCr levels in the soleus muscle and the circulating blood concentrations of 17β-estradiol. These findings provide in vivo evidence supporting the additional role of 17β-estradiol in modulating the energy metabolism of skeletal muscle. These insights may have clinical significance in the management of symptoms associated with estrogen deficiency in females. Furthermore, our work highlights the need for further research with larger cohorts and a broader scope to fully elucidate the complex interplay of hormones and energy metabolism in skeletal muscle.

## Figures and Tables

**Table 1:** ^31^P MRS measurements of the metabolite-to-ATP ratio (by integral), intracellular pH, and free [Mg^2+^] (mM) in the soleus muscle of young females (*n* = 7).

			Metabolite-to-ATP Ratio (a.u.)			
	δ (ppm)			EF		PO
PME	6.63	± 0.21	0.10	± 0.04	0.07	± 0.03
Pi(ex)	5.14	± 0.06	0.06	± 0.03	0.05	± 0.02
Pi(in)	4.81	± 0.02	0.30	± 0.08	0.31	± 0.09
GPE	3.51	± 0.04	0.03	± 0.01	0.03	± 0.01
GPC	2.97	± 0.01	0.19	± 0.10	0.20	± 0.10
PCr	[0]		3.82	± 0.18	4.05	± 0.18 *
γ-ATP	−2.41	± 0.01	[1.0]		[1.0]	
α-ATP	−7.48	± 0.02	1.08	± 0.08	1.09	± 0.08
NAD	−8.06	± 0.10	0.27	± 0.05	0.33	± 0.14
β-ATP	−15.95	± 0.03	1.16	± 0.07	1.25	± 0.08
pH			6.983	± 0.014	6.990	± 0.018
Mg (mM)			0.68	± 0.08	0.60	± 0.08

Note: Data were measured with 2D ^31^P MRSI at 7T under conditions of TR = 1 s and TE = 0.5 ms using a partial volume T/R RF coil; PCr was used as an endogenous reference for the chemical shift at 0 ppm and γ-ATP as a reference for signal intensity (integral). Gaussian lineshape deconvolution was performed between intra- and extracellular Pi and between α-ATP and NAD. Signal intensity was reported without correction for partial saturation. * indicates *p* < 0.05.

**Table 2:** Results of the linear correlation between 17β-estradiol and PCr.

Metabolites	*p*-Value	r-Value
PME	0.951	−0.018
Pi(ex)	0.985	−0.005
Pi(in)	0.332	0.280
GPE	0.625	−0.143
GPC	0.766	0.088
PCr	0.014 *	0.638
γ-ATP	-	−
α-ATP	0.295	0.301
NAD	0.458	−0.216
β-ATP	0.158	0.398
pH	0.910	0.033
Mg	0.290	−0.304

Note: 17β-estradiol levels (pg/mL) were measured in serum, PCr signal intensities were measured from the soleus muscle in reference to γ-ATP by integral; intracellular pH was measured from the Pi(in) chemical shift with respect to PCr by Equation (1); and the free Mg^2+^ concentration (in mM) was measured from the β-ATP chemical shift by Equation (2). * indicates *p* < 0.05.

## Data Availability

The datasets presented in this article are not readily available because the data are part of an ongoing study. Requests to access the datasets should be directed to Jimin Ren.

## Abbreviations

ADP	adenosine diphosphate
BMS	bulk magnetic susceptibility effect
Cr	free creatine
EF	early follicular
ER	estrogen receptors
GPC	glycerolphosphocholine
GPE	glycerolphosphoehtanolamine
HF	header-foot
MPL	membrane phospholipids
NAD	nicotinamide adenine dinucleotide, a combination of NAD^+^ and NADH
NTP	nucleoside triphosphates
PCr	phosphocreatine
PC	phosphocholine
PE	phosphoethanolamine
Pi	inorganic phosphate
PDE	phosphodiester
PME	phosphomonoester
PO	peri-ovulation
PTH	parathyroid hormone
